# Rebaudioside affords hepatoprotection ameliorating sugar sweetened beverage- induced nonalcoholic steatohepatitis

**DOI:** 10.1038/s41598-020-63688-z

**Published:** 2020-04-21

**Authors:** Dong Xi, Jashdeep Bhattacharjee, Rosa-Maria Salazar-Gonzalez, Soyoung Park, Alice Jang, Mikako Warren, Russell Merritt, Sonia Michail, Sebastien Bouret, Rohit Kohli

**Affiliations:** 1 Gastroenterology, Hepatology and Nutrition, 90027, Los Angeles, CA USA; 2 Developmental Neuroscience Program & Diabetes and Obesity Program, Center for Endocrinology, Diabetes and Metabolism, 23298, Richmond, VA USA; 3 Pathology and Laboratory Medicine, Children’s Hospital Los Angeles & University of Southern California Keck School of Medicine, Los Angeles, CA 90027 USA

**Keywords:** Non-alcoholic fatty liver disease, Microbiota

## Abstract

Sugar-sweetened beverage consumption is a known independent risk factor for nonalcoholic steatohepatitis (NASH). Non-caloric sweeteners (NCS) are food additives providing sweetness without calories and are considered safe and/or not metabolized by the liver. The potential role of newer NCS in the regulation of NASH, however, remain unknown. Our study aimed to determine the impact of newer NCS including Rebaudioside A and sucralose on NASH using high fat diet induced obesity mouse model by substituting fructose and sucrose with NCS in the drinking water. We characterized the phenotype of NCS- treated obesity and investigated the alterations of hepatic function and underlying mechanisms. We found that NCS have no impact on weight gain and energy balance in high fat diet induced obesity. However, in comparison to fructose and sucrose, Rebaudioside A significantly improved liver enzymes, hepatic steatosis and hepatic fibrosis. Additionally, Rebaudioside A improved endoplasmic reticulum (ER) stress related gene expressions, fasting glucose levels, insulin sensitivity and restored pancreatic islet cell mass, neuronal innervation and microbiome composition. We concluded that Rebaudioside A significantly ameliorated murine NASH, while the underlying mechanisms requires further investigation.

## Introduction

Current treatment strategies for nonalcoholic steatohepatitis (NASH) have focused on lifestyle management of modifiable risk factors, through a combination of diet and exercise^1–3^. Potential therapeutic targets for NASH intersect with the complex pathogenesis of NASH including hepatic steatosis from the imbalance of lipogenesis and free fatty acid (FFA) promoting inflammatory response and fibrosis progression^4,5^. However, despite pharmaceutical agents currently in advanced stages of clinical testing^6–8^, NASH is on track predicted to become the main reason for liver transplant in the very near future^9^. Therefore, it is essential to continue to explore novel NASH therapies.

Sugar sweetened beverages are now well acknowledged to have severe consequences on human health. Consequently, non-caloric sweeteners (NCS) such as aspartame, sucralose, saccharin, and Rebaudioside A have increased in popularity and usage. However, there is a limited evidence for the benefits of frequent consumption of NCS sugar substitutes. This is especially true for the most recent addition Rebaudioside A, that is an extract of the stevia leaf that provides sweetness without calories^10^. Interestingly, recent literature has reported that Rebaudioside A may in fact play a role in glucose metabolism and has even been reported to improve post-prandial glucose-insulin index^11^, and its consumption may result in weight loss in mice fed a high fat diet^12^. These observations suggest a potential role for Rebaudioside A on glucose metabolism in general, and on liver function and NASH in particular.

The well-known interactions between human health, diet and intestinal microbiota are based on the involvement of the microbiome in metabolism and immunity, which also participates in the pathogenesis of NASH^13,14^. The composition and function of microbiome are modulated rapidly by diet, such as fermentation of undigested carbohydrates^15,16^. Interestingly Everard *et al*. recently reported that Akkermansia muciniphila partially counteracts obesity and metabolic comorbidities^17^. Transplantation of fecal contents from saccharin fed mice into germ-free mice was reported to transfer the glucose tolerance phenotype to recipient mice, thus highlighting the potential role of the microbiome metabolic changes secondary to NCS in diet^18^. As Rebaudioside A is not absorbed from the intestine^19^, the role of the microbiome may be further important to explain any improved metabolic outcomes observed.

Thus, we performed experiments to further understand the role of NCS in metabolic outcomes^20,21^ with a focus to understand the effects of NCS on glucose metabolism, liver function and changes in the microbiome.

## Results

### NCS has no impact on weight gain and adiposity, and energy balance in diet induced obesity

**Figure 1:**
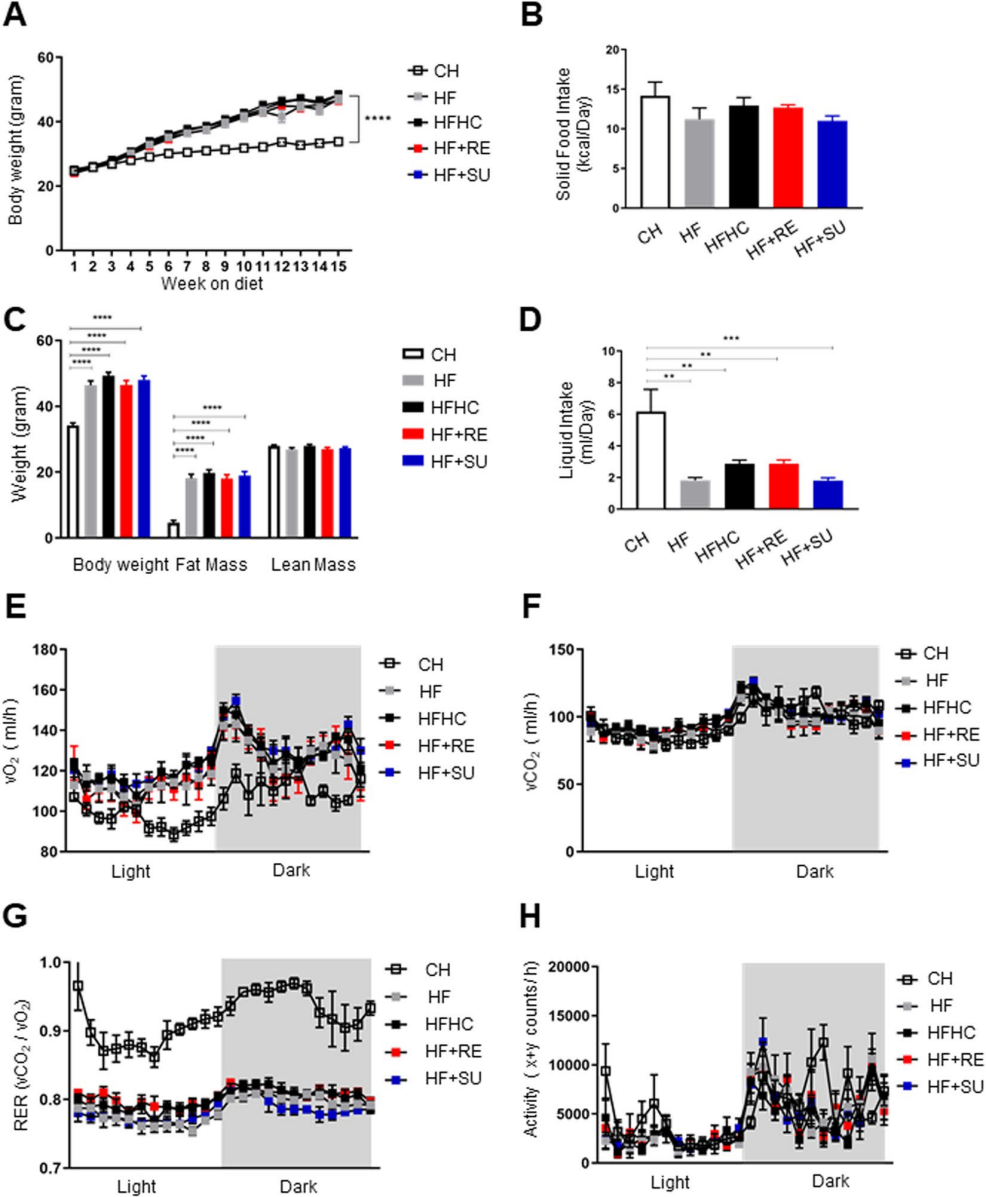
NCS has no impact on weight gain and adiposity, and energy balance in high fat diet- induced obesity. NCS did not change the effects of high fat diet on body weight (**A**), solid food intake (**B**) and liquid intake (**D**), body composition (**C**), as well as activity and energy expenditure (**E**–**H**), compared to fructose and sucrose, while compared to CH group, all four high fat diet- fed groups demonstrated significant increased weight gain and decreased resting energy expenditure. (****p < 0.0001; ***p < 0.001; **p < 0.01; *p < 0.05 indicates statistical difference from comparison between CH group and high fat diet- fed groups).

To investigate the effects of NCS on obesity, we characterized the phenotype of the WT type mice that received high fat diet but different drinking water containing fructose and sucrose, or Rebaudioside A or sucralose. Compared to CH group (33.8 g ± 0.4), we observed all four groups fed on high fat diet have gained body weight significantly (p < 0.0001), however we observed no significant difference in body weight gain among the high fat diet fed groups at 15 weeks of the study (p > 0.05) (HF group: 47.0 g ± 1.2; HFHC group: 48.3 g ± 1.3; HF + RE group: 46.8 g ± 1.3; HF + SU group: 48.2 g ± 1.0), indicating NCS has no impact on weight gain while mice have access to a high fat diet (Fig. 1A). Body composition analysis revealed that no significant difference of lean mass among all 5 groups (p > 0.05) (Lean mass: CH group: 28.0 g ± 0.3; HF group: 26.9 g ± 0.6; HFHC group: 27.9 g ± 0.5; HF + RE group: 26.9 g ± 0.6; HF + SU group: 27.3 g ± 0.4); while the 4 groups that received a high fat diet demonstrated similar amount of fat mass (p > 0.05) (HF group: 18.1 g ± 1.2; HFHC group: 19.7 g ± 1.0; HF + RE group: 18.1 g ± 1.2; HF + SU group: 19.0 g ± 1.2), but higher than CH group (4.7 g ± 0.6) (p < 0.05) (Fig. 1C).

To investigate the relationship between body weight gain, caloric intake and energy expenditure, we performed metabolic studies for 5 consecutive days on 4 representative mice for each group No difference in caloric intake was observed among all the groups, despite being given either chow diet or high fat diet, and different drinking water (Fig. 1B; Supplementary Table 1), suggesting that the macronutrient composition of the diet, rather than the calorie content of the diet, led to obesity. The liquid intake of the CH group (6.2 ml ± 1.4) was significantly higher than the other 4 groups receiving a high fat diet (HF group: 1.8 ml ± 0.2; HFHC group: 2.9 ml ± 0.2; HF + RE group: 2.9 ml ± 0.2; HF + SU group: 1.8 ml ± 0.2 ml) (p < 0.05) (Fig. 1D). Oxygen consumption and CO_2_ production were found to be not significantly different among all 5 groups, as well as the level of activity (p > 0.05; Fig. 1E,F,H). However, Respiratory exchange ratio (RER) of CH group was higher than the 4 high fat diet- fed groups (p < 0.05) (Fig. 1G).

### Rebaudioside A ameliorates diet- induced liver dysfunction and hepatic steatosis

**Figure 2:**
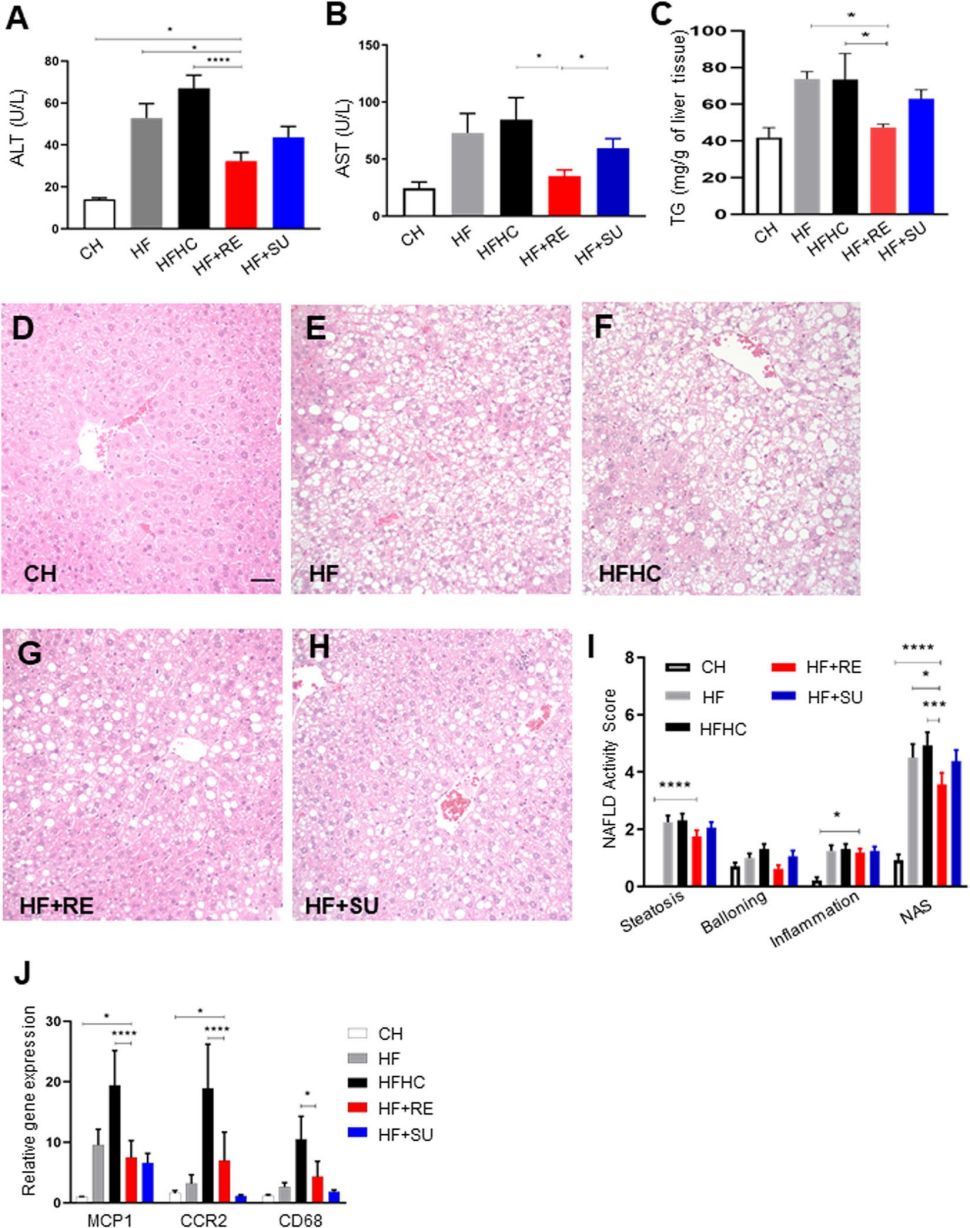
Rebaudioside A ameliorates diet- induced liver dysfunction and hepatic steatosis. Rebaudioside A improved liver enzymes (**A**,**B**), triglyceride level (**C**) and NAFLD activity score (**D**–**I**) and inflammation (**J**), compared to fructose and sucrose. (****p < 0.0001; ***p < 0.001; **p < 0.01; *p < 0.05 indicates statistical difference from comparison between HF + RE and CH, HF, HFHC, HF + RE groups).

We investigated the effects of NCS on NASH by measuring liver enzymes and examining hepatic histology. Interestingly, alanine aminotransferase (ALT) of HF + RE group (60.7 ± 7.7 U/L) was found to be significantly lower than HF group (73.3 ± 11.3 U/L) and HFHC group (87.2 ± 11.4 U/L) (p < 0.05), while not significantly different from CH group (40.4 ± 5.9 U/L) (Fig. 2A). Mice of HF + RE (35.0 ± 5.6 U/L) group have significantly lower plasma Aspartate Aminotransferase (AST) than in comparison to mice of HFHC (84.6 ± 19.4 U/L, p < 0.05) and HF + SU (59.6 ± 8.3 U/L, p < 0.05) however mice of HF group have no significant difference in plasma AST (72.9 ± 17.1 U/L) (Fig. 2B) suggesting that Rebaudioside A may improve liver injury from a high fat diet. NAFLD activity score (NAS) was utilized for histologically scoring of the liver. We found that Rebaudioside A significantly decreased the triglyceride (Tg) level (Fig. 2C), as well as NAFLD activity score (NAS) (3.79 ± 0.05) compared to HF (4.29 ± 0.06) and HFHC groups (4.71 ± 0.06) (p < 0.05), by improving the severity of hepatic steatosis (1.85 ± 0.03 in HF + RE group versus 2.14 ± 0.03 in HF group, 2.29 ± 0.03 in HFHC group, p < 0.05) and ballooning (0.71 ± 0.01 in HF + RE group versus 0.93 ± 0.02 in HF group, 1.29 ± 0.02 in HFHC group, p < 0.05) (Fig. 2D–I). We further observed that the HF + RE group had lower expression of hepatic inflammatory genes including, monocyte chemoattractant protein 1 (MCP1: 7.49 ± 2.80 (HF + RE) vs 19.40 ± 5.76 (HFHC); p < 0.05), C-C chemokine receptor type 2 (CCR2: 7.00 ± 4.69 (HF + RE) vs 18.90 ± 7.31 (HFHC); p < 0.05), and Cluster of Differentiation 68 (CD68: 4.35 ± 2.53 (HF + RE) vs 10.50 ± 3.78 (HFHC); p < 0.05) in comparison to the HFHC group (Fig. 2J), but not against the HF group. No beneficial effect of sucralose was demonstrated in HF + SU group with the NAS of 4.36 ± 0.05 (p > 0.05).

### Rebaudioside A induces less hepatic fibrosis and ER stress than HFHC

**Figure 3:**
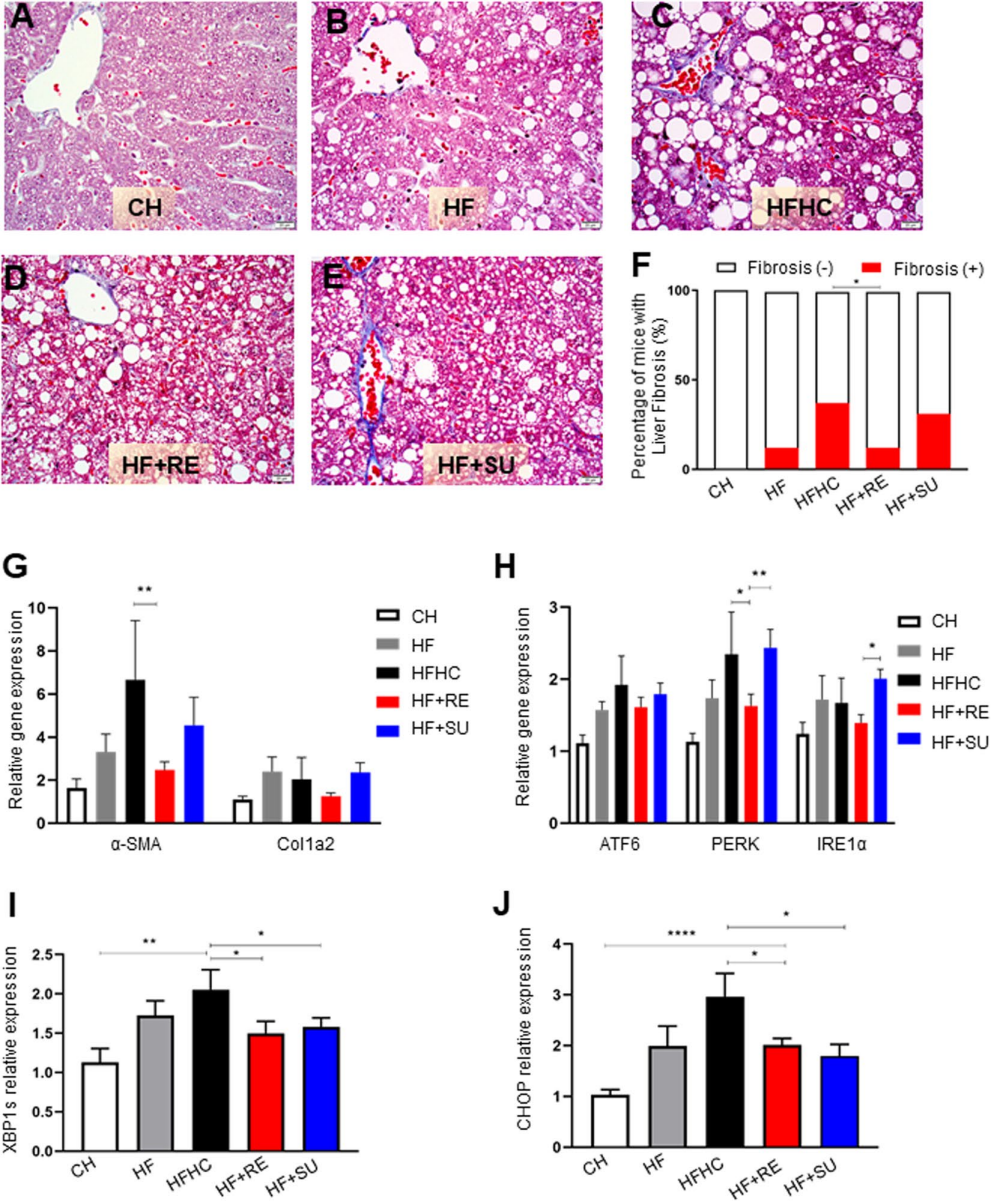
Rebaudioside A induces less hepatic fibrosis and ER stress than HFHC. Rebaudioside A (**D**) ameliorated the risk of developing fibrosis from the analysis of trichrome staining, compared to fructose and sucrose (**C**,**F**). HFHC-induced upregulation of mRNA expression of α-SMA, Col1A2 (**G**);ATF6, PERK, IRE1α (**H**); XBP1s (**I**) and CHOP (**J**) was ameliorated by Rebaudioside A. (****p < 0.0001; ***p < 0.001; **p < 0.01; *p < 0.05 indicates statistical difference from comparison between HF + RE and CH, HF, HFHC,HF + RE groups).

To investigate the distinct effects of NCS on hepatic fibrosis from HFHC, we utilized trichrome staining and qPCR to examine the protein and mRNA expression of fibrosis related biological markers. Histologically the risk of fibrosis was analyzed by the percentage of mice developing fibrosis in each group using trichrome and Sirius Red staining. We observed that Rebaudioside A decreased presence of fibrosis to 12% compared to 37% in HFHC and HF + SU groups (p < 0.05) (Fig. 3A–F; Supplementary Fig. 2), while similar to HF group at 12%. At the molecular level, we found that compared to CH group, mRNA expression of both α-Smooth Muscle Actin (α-SMA) and Collagen Type I Alpha 2 Chain (Col1A2), the fibrosis related genes, was significantly elevated in HF (α-SMA, 3.3 ± 0.8; Col1A2, 2.4 ± 0.7) and HFHC groups (α-SMA, 6.6 ± 2.7; Col1A2, 2.0 ± 1.0) (p < 0.05) (Fig. 3G), while this upregulation was ameliorated by Rebaudioside A (α-SMA, 2.5 ± 0.4, p < 0.05 compared to HFHC; Col1A2, 1.3 ± 0.1). Since disruption of intracellular homeostasis leads endoplasmic reticulum (ER) stress which is associated with pro-apoptotic signaling and fibrotic diseases, we further explored the mechanic role of ER stress in Rebaudioside A-induced hepatoprotection. Interestingly, we found that mRNA expression of ER stress related genes was significantly increased in HFHC group compared to CH, including Activating transcription factor 6 (ATF6) (1.9 ± 0.4), PRKR-like endoplasmic reticulum kinase (PERK) (2.4 ± 0.6) and Inositol-Requiring Enzyme 1 α (IRE1α) (1.7 ± 0.3) (p < 0.05), however this change was successfully reversed by Rebaudioside A (ATF6, 1.6 ± 0.1), PERK (1.6 ± 0.2) and IRE1α (1.4 ± 0.1) (p < 0.05) (Fig. 3H), as well as spliced form of X-box binding protein 1 (XBP1s), CCAAT-enhancer-binding protein homologous protein (CHOP) (p < 0.05) (Fig. 3I,J) and the protein expression of CHOP (Supplementary data and Supplementary Fig. 3). These phenomena were not observed with the administration of sucralose in HF + SU group compared to HFHC (p > 0.05). It suggested that HFHC-induced excessive ER stress was partially inhibited by Rebaudioside A.

### Rebaudioside A improves indices of glucose homeostasis and insulin sensitization

**Figure 4:**
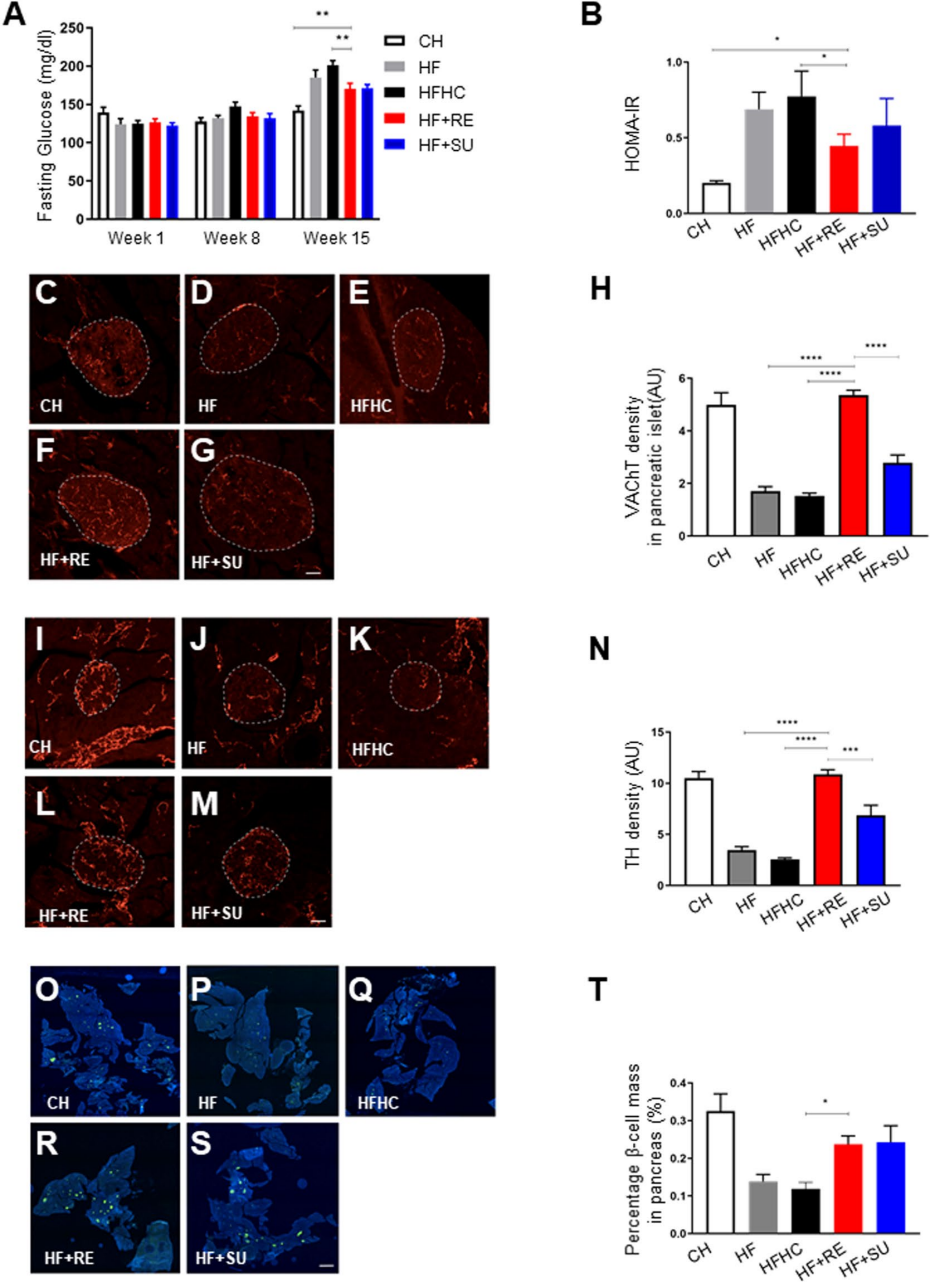
Rebaudioside A improves indices of glucose homeostasis, beta cell mass, autonomic innervation. NCS improved fasting glucose level (**A**) and restored pancreatic VAChT (**C**–**H**), TH density (**I**–**N**), and beta cell mass (**O**–**T**) compared to fructose and sucrose, while Rebaudioside A specifically improved insulin sensitivity (**B**). (****p < 0.0001; ***p < 0.001; **p < 0.01; *p < 0.05 indicates statistical difference from comparison between HF + RE and CH, HF, HFHC, HF + RE groups).

Glucose intolerance, insulin resistance, and diabetes are all considered to be either underlying mechanism(s) and/or important comorbidities for NASH. We measured the fasting glucose levels pre-intervention and 8 weeks, 15 weeks post-intervention to assess the effects of different diets and water. We found that the HF + RE group demonstrated a significantly decreased fasting glucose level at 15 weeks post-intervention (170 ± 1.3) compared to HF (185 ± 1.8) and HFHC groups (201 ± 1.0) (p < 0.05), and similar trend was observed in HF + SU group (Fig. 4A). Moreover, levels of HOMA-IR, a marker of beta cell function and insulin resistance, were also significantly reduced in the HF + RE group (Fig. 4B). The autonomic nervous system plays a critical role in the regulation of glucose homeostasis. In particular, parasympathetic neurons that stimulate the secretion of insulin densely innervate the islets of Langerhans^22,23^. We therefore analyzed the autonomic innervation of pancreatic islets by performing immunostaining for vesicular acetylcholine transporter (VAChT), which is a marker for parasympathetic neurons. The density of VAChT-immunoreactive fibers innervating pancreatic beta cells was significantly reduced in HF and HFHC groups compared to the CH group. Remarkably, the density of VAChT fibers in HF + RE was similar to the CH control group, and the HF + SU group displayed slightly improved VAChT innervation (Fig. 4C–H) and tyrosine hydroxylase (TH) density compared to HF and HFHC animals (p < 0.05; Fig. 4I–N). Pancreatic islet mass was also reduced in HF and HFHC animals compared to CH mice, but both the HF + RE and HF + SU groups displayed increased pancreatic beta cell mass (p < 0.05; Fig. 4O–T); the density of TH labeled fibers was improved 3-fold in HF + RE and 1.5-fold in HF + SU fed mice compared to HF mice (p < 0.05; Fig. 4I–N). Together, these data preliminarily imply that Rebaudioside A and sucralose enhance autonomic innervation of pancreas islets and improve beta cell mass in a high fat diet induced obesity model, while requires further investigation.

### Rebaudioside A induces distinct changes to microbiome composition from HFHC

**Figure 5:**
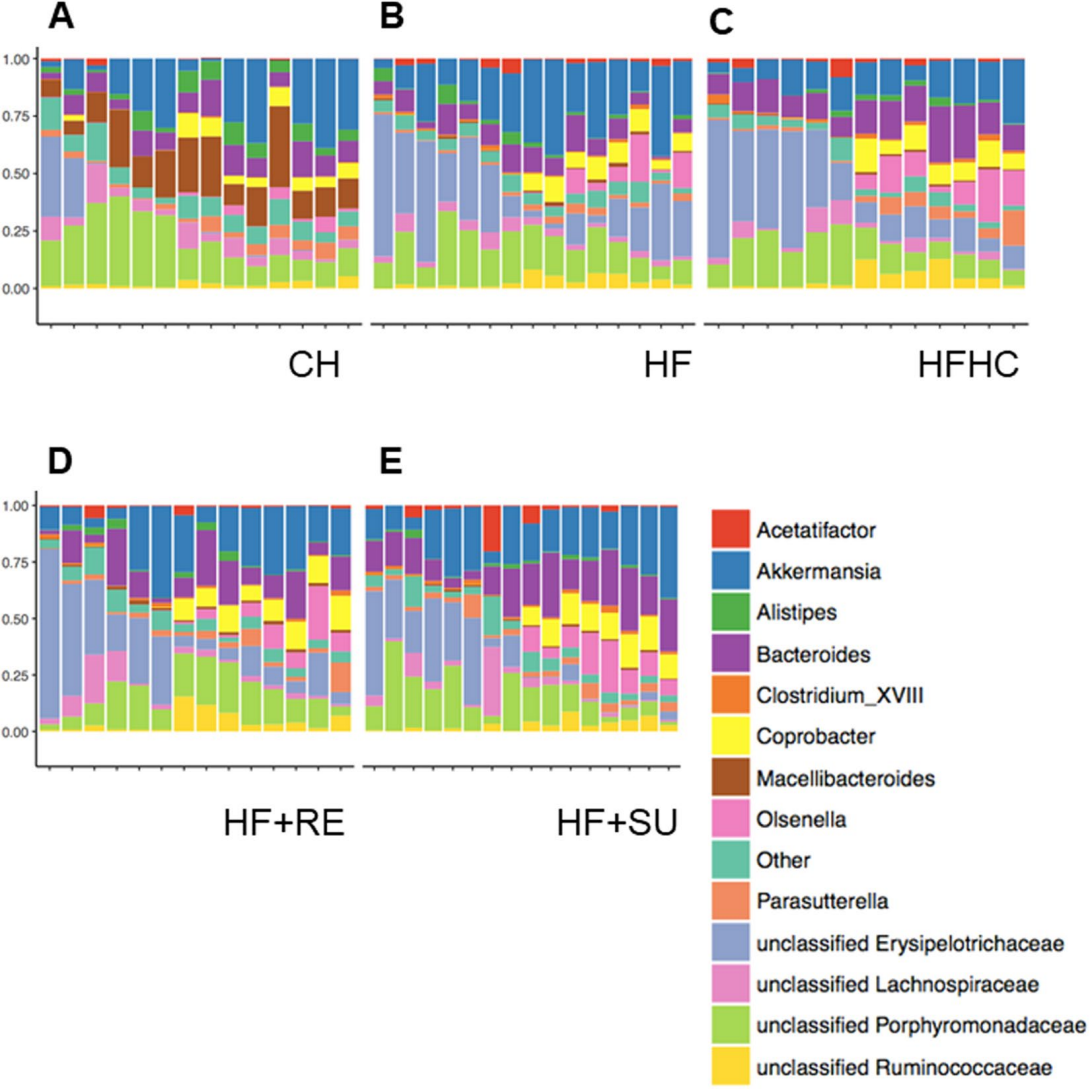
Rebaudioside A induces distinct changes to microbiota composition from HFHC. Analysis of microbiota data in each group was shown in (**A**–**E**), including the distribution of the 14 representative abundant microbes. Rebaudioside A (HF + RE), compared to HFHC, relatively increased the composition of Akkermansia, while decreased the composition of Bacteroides.

The distribution of representative abundant microbes was analyzed statistically (Fig. 5). We observed that between Rebaudioside A in HF + RE group and HFHC, Akkermansia and Bacteroides demonstrate most variable numbers, suggesting their potential regulation by different diets and drinking water. However, statistical analysis of two individual species showed no difference between these two groups (p > 0.05) (Data not shown). This is consistent with previous studies that reported that the number of individual species is variable between individuals and that no individual bacterial species can explain the complex mechanism of NASH. We then found that the ratio between Akkermansia and Bacteroides was higher in HF + RE group on Rebaudioside A (2.27 ± 0.9) than HFHC group (1.14 ± 0.7) (Data not shown). It suggests a potential mechanistic role of the ratio between certain species in the pathogenesis of NASH.

## Discussion

Varied findings have been reported regarding the effects of various NCS on human metabolism and liver function^24–29^. On the one extreme some studies have reported that consumption of NCS by women during pregnancy may result in development of metabolic syndrome in their children^30,31^. Our data, on the other hand, suggest that that replacement of fructose and sucrose with Rebaudioside A as a sweetener, may provide hepatoprotection. We report data that Rebaudioside A usage is associated with improved glucose tolerance, less liver fibrosis and inflammation mediated via decreased ER stress. Thus, our novel findings suggest that Rebaudioside A maybe a potential “metabolically healthy” NCS agent and present an opportunity to study the underlying mechanisms of this metabolic-protection afforded by Rebaudioside A consumption.

For example, Rebaudioside A may have the potential to inhibit hepatic oxidative stress and NFκβ mediated inflammatory response by upregulation of nuclear factor erythroid 2–related factor 2 (Nrf2)^32^. We speculate this as a potential mechanism given data from *in vitro* studies wherein Rebaudioside A was reported to not be absorbed by intestinal cells but rather degraded by the microbiome^33,34^. Further, it was reported that Rebaudioside A protected human hepatocytes in culture (HepG2 cells) from oxidative stress induced by carbon tetrachloride and inhibited development of fibrosis^35^. These findings are consistent with our observation that Rebaudioside A improves sugar (fructose & sucrose) sweetened water induced obesity associated hepatic steatosis and hepatic fibrosis.

Though various mechanisms were proposed, hepatic fibrosis always involves excessive deposition of collagen and extracellular matrix proteins from activated myofibroblasts which express α-SMA^36^. Our study also confirmed the elevation of fibrosis related gene expression, including αSMA and Col1A2 in HFHC-induced hepatic fibrosis. ATF6^37^, PERK^38^ and IRE1α^39^ are well characterized elements of the ER stress response. ER stress in turn can be associated with liver fibrosis^40^. Our finding that the inhibition by Rebaudioside A on the HFHC- activated three pathways for ER stress could revert the profibrotic phenotype of activated myofibroblasts in patients with NASH.

Our research reports that Rebaudioside A, as a type of NCS, appears to downregulate fasting glucose levels and upregulate insulin sensitivity. These effects were achieved possibly through restoring both autonomic nerve innervation and the cell mass of pancreatic islets. Our data suggest that Rebaudioside A- induced hepatoprotection on NASH is at least associated with, or even mediated by, improved pancreatic endocrine function and insulin sensitivity.

Recent studies have reported that consuming NCS may disrupt microbiome diversity in both rats and humans^18,41–44^ and further suggest that this may lead to glucose intolerance. Similarly, another cross-sectional study reported that consuming NCS has no effect on microbiome abundance and gene function however consuming NCS alters the microbiome diversity^45^. Further studies have also shown that alteration of microbial diversity may lead to metabolic changes^46,47^. Specifically, it has been reported that Bacteroides perform efficient hydrolysis of Rebaudioside A to steviol^34^ and there are even reports of an inverse relation with abundance of Akkermansia and the body weight of mice and humans^48,49^. Our study preliminarily found that the ratio between Akkermansia and Bacteroides was potentially improved by Rebaudioside A as compared to HFHC, which is intriguing and can be a potential focus of future research.

NASH is predicted to be a leading cause of transplantation for both pediatric and adult liver diseases in the near future^9^, therefore it becomes essential and important to identify its pathological mechanisms and therapeutic targets, other than lifestyle changes. Our study for the first-time reports that Rebaudioside A improves diet-induced NASH and hepatic fibrosis, possibly through improving ER stress, insulin sensitization, pancreatic autonomic innervation and microbiome composition. Considering that are only a few human microbiota studies in NASH demonstrating an association between gut dysbiosis and NASH, further investigation is warranted regarding effects of Rebaudioside A on microbiome diversity in NASH, and the underlying molecular mechanisms.

## Materials and Methods

### Ethics statement

All procedures were carried out in accordance with the National Institutes of Health Guidelines on the Care and use of Animals and approved by Children’s Hospital Los Angeles Institutional Animal Care and Use Committee (IACUC) (Protocol 392)

### Experimental schedule

6~8-week-old wild-type (WT) mice were randomly divided into 5 groups (N = 16 for each group) and received different diets and drinking water for 15 consecutive weeks until sacrificed. The description of different groups is as follows: chow group (CH group) was placed on a chow diet (PicoLab® Rodent Diet 20, LabDiet, St.Louis, MO) with regular water; high fat group (HF group) was placed on a high fat diet (Rodent Diet With 58 kcal% Fat and Sucrose, Research Diet. New Jersey, USA) with regular water; high fat high carbohydrate group (HFHC group) was placed on a high fat diet and water containing sucrose (Acros Organic, New Jersey, NY), fructose(Acros Organic, New Jersey, NY); Rebaudioside A group (HF + RE) was placed on a high fat diet and water containing Rebaudioside A (Sigma, St. Louis, MO); sucralose group (HF + SU) was placed on a high fat diet and water containing sucralose (Sigma, St. Louis, MO). The sweetness of different water containing sucrose and fructose, or Rebaudioside A, or sucralose is equivalent, including 23.1 g/L fructose and 18.9 g/L sucrose for HFHC group, 194 mg/L Rebaudioside A for HF + RE group and 97 mg/L sucralose for HF + SU group. Body weight was measured weekly. At 15 weeks of the study, food and water intake, O2 and CO2 production, energy expenditure, respiratory exchange ratio (i.e., VCO2/O2), and locomotor activity (XY) were monitored using a combined indirect calorimetry system (TSE Systems). The mice were acclimated in monitoring chambers for 2 days, and the data were collected for 3 days. In addition, body composition analysis (fat/lean mass) was measured using NMR (EchoMRI, Texas, USA). These metabolic measures were performed at the Rodent Metabolic Core of Children’s Hospital Los Angeles. Mice were then anesthetized, and liver, pancreas, plasma and microbiome samples were collected.

### Hepatic triglyceride and serum ALT assay

Hepatic triglyceride (Tg) content was estimated by extracting lipid from the liver using a chloroform free lipid extraction kit^50^ (ABCAM, Cambridge, United Kingdom) as per manufacturer’s instruction. Briefly, 50 mg of liver tissue was homogenized in 500 µl of Extraction buffer (provided with the lipid extraction kit). The homogenate was centrifuged at 10,000 g for 5 minutes at 4 °C and supernatant was collected in a clean tube. The supernatant was agitated on orbital shaker for 20 minutes at room temperature followed by centrifugation at 10,000 g for 5 minutes at 4 °C. The supernatant was collected in a fresh tube and the volume of supernatant was noted. The cap of the tube containing supernatant was left open in a dry incubator maintained at 37 °C overnight. The lipid extract was suspended using 50 µl of Suspension buffer (provided with the lipid extraction kit). The suspended lipid extract was sonicated for 20 minutes at 37 °C. 2 µl of the lipid extract per sample was mixed with 200 µl of enzymatic solution provided in the Triglyceride (GPO Liquid Reagent Set; Pointe Scientific, Canton, MI) as per manufacturer’s instructions incubated at 37 °C for 5 minutes followed by measuring colorimetric absorbance at 500 nm to determine the hepatic Tg content (BioTek, Winooski, VT). ALT was measured from the serum collected with DiscretPak ALT Reagent Kit (Catachem, Bridgeport, CT). The enzyme level was determined over a 5-minute interval by measuring the photometric absorbance at 340 nm.

### Quantitative PCR

250 ng of total RNA was used to generate cDNA for each sample using Reverse Transcription Master Mix (Fluidigm, San Francisco, CA). The cDNA was pre-amplified for 14 cycles with the primers of DELTAgene Assay (Fluidigm, San Francisco, CA) in a multiplex PCR reaction using PreAmp Master Mix (Fluidigm, San Francisco, CA). The pre-amplified PCR products were treated with Exonuclease I (New England Biolabs, Ipswich, MA). The pre-amplified Exonuclease treated products was diluted 5-fold using TE buffer (TEKnova, Hollister, CA) and used as sample in the Sample inlets of primed Dynamic Array 96.96 GE IFC (Fluidigm, San Francisco, CA). 5 μM concentration of each primer of DELTAgene Assay was added to Assay inlets of primed Dynamic Array 96.96 GE IFC, according to manufacturer’s instruction. The real-time PCR data was collected using the Biomark HD (Fluidigm, San Francisco, CA) with the instrument settings as specified by the manufacturer. Each sample had 4 replicates. The fold change of gene expression was calculated using Fluidigm Real-Time PCR Analysis software (Fluidigm, San Francisco, CA).

### Taqman assay

1 µg of total RNA isolated from liver of the mice incubated with DNAse1 (Invitrogen, Carlsbad, CA) at room temperature for 15 minutes followed by inactivation of DNase1 activity by addition of 25 mM of EDTA incubated at 65oC for 10 minutes. SuperScript™ III First-Strand Synthesis System (Invitrogen, Carlsbad, CA) was used to prepare the cDNA for the total RNA according to manufacturer’s product data sheet. Taqman probe for Rpl18 (Applied Biosystems, Foster City, CA), XBP1 spliced variant of (Xbp1s) (Applied Biosystems, Foster City, CA) and CHOP (Applied Biosystems, Foster City, CA) used to determine the expression Xbp1s and CHOP on 7900HT qPCR platform (Applied Biosystems, Foster City, CA). ΔΔCt was used to calculate the fold change of expression of Xbp1s and CHOP normalized with housekeeping gene, Rpl18.

### Immunohistochemistry

Liver tissue was first fixed in 10% formalin for 48 hours, then subjected to microtome sectioning to generate 5 um thick sections for immunohistochemistry, which was performed by pathology research core at Children’s Hospital Los Angeles. Hematoxylin & Eosin (H&E) staining was performed on the sections. Histology was read by a single independent pathologist blinded to the experimental design. Histological scoring of the liver was performed using the NAS system, including the score of hepatic steatosis (0–3), inflammation (0–3) and ballooning (0–2). Fibrosis was then evaluated by trichrome and Sirius Red staining.

Pancreatic tissue was dissected and fixed in 4% paraformaldehyde overnight. Tissue were then frozen, sectioned at 20-um thickness, and processed for immunofluorescence using standard procedures^51,52^. The primary antibodies used for IHC were as follows: guinea pig anti-insulin (1:500, Abcam), rabbit anti-vesicular acetylcholine transporter (VAChT, 1:500, Synaptic Systems), and mouse anti-tyrosine hydroxylase (TH, 1:4000, immunostar). The primary antibodies were visualized with Alexa Fluor 568 donkey anti-mouse IgG, Alexa Fluor 488 donkey anti-guinea pig IgG and Alexa Fluor 568 donkey anti-rabbit IgG (1:200, Millipore). Sections were counterstained with bis-bezamide (1:10,000, Invitrogen) to visualize cell nuclei. For the quantitative analysis of VAChT fiber density in insulin+ pancreatic islets, and beta cell mass, images were acquired using a Zeiss LSM 710 confocal system equipped with a 20X objective through the pancreas. The average number of cells and density of fibers were analyzed in 2-4 sections per animal with totally 6 mice in each group (N = 6). The image analysis was performed using ImageJ analysis software (NIH). Briefly, each image plane was binarized to isolate labeled fibers from the background and to compensate for differences in fluorescence intensity. The integrated intensity, which reflects the total number of pixels in the binarized image, was then calculated for each image as previously described^51,52^. This procedure was conducted for each image plane in the stack, and the values for all of the image planes in a stack were summed.

### Microbiome analysis

DNA from stool samples was isolated on a Qiagen Qiacube using Lysing Matrix E Bead tubes (MP Biomedicals #116914050) and the Qiagen AllPrep DNA/RNA Mini Kit (Qiagen #80204).

The 16S bacterial DNA region from stool DNA and negative controls were amplified by PCR using a shared forward primer 806 rB (CAAGCAGAAGACGGCATACGAGATAGTCAGCC-

AGCCGGACTACNVGGGTWTCTAAT) for all samples, while each sample had its own unique identifying reverse primer^53^, which were modified from the original 515F-806R primer pairs^54^. Each sample was done in triplicate PCR wells and mixed together for final pooling. Library qualities were accessed on Agilent High Sensitivity DNA Bioanalyzer chips. All samples were pooled and sequenced using custom sequencing primers; R1 (TATGGTAATTGTGTGYCAGCMGCCGCGGTAA), R2 (AGTCAGCCAGCC- GGACTACNVGGGTWTCTAAT) and Index (AATGATACGGCGACCACCGAGATCT- ACACGCT). Paired-end sequencing (2 × 150 bp) using Illumina MiSeq Reagent Kit v2 flowcell was performed on an Illumina MiSeq System.

Reads were de-multiplexed using QIIME v1.9.1 and then processed to generate an amplicon sequence variant (ASV) table using DADA2 v1.5.2. Briefly, DADA2 performs quality filtering, de-noising, sample inference, and chimera removal using an empiric error model to generate exact amplicon sequences. Taxonomies were assigned to each sequence variant using the RDP naïve Bayes classifier. Statistical analyses were performed using the ‘phyloseq’ (v1.20.0) package in the R statistical environment.

### Statistical analysis

Statistical analysis was performed using t-test, Chi-square or two-way ANOVA followed by Bonferroni post-hoc test. P value of <0.05 was considered statistically significant. Results were presented as mean ± SEM.

## Supplementary information

Supplementary information.
