# Supplementary material for: Rebaudioside affords hepatoprotection ameliorating sugar sweetened beverage- induced nonalcoholic steatohepatitis
Source: Sci Rep. 2020 Apr 21;10:6689. doi: 10.1038/s41598-020-63688-z (PMC7174355; doi:10.1038/s41598-020-63688-z)
Supplement: Supplementary file 1 — Supplementary information. [file 41598_2020_63688_MOESM1_ESM.docx]

Rebaudioside affords hepatoprotection ameliorating sugar sweetened beverage- induced nonalcoholic steatohepatitis

Dong Xi ^1#^, Jashdeep Bhattacharjee ^1#^, Rosa-Maria Salazar-Gonzalez ^1^, Soyoung Park ^2^, Alice Jang ^2^, Mikako Warren ^3^, Russell Merritt ^1^, Sonia Michail ^1^, Sebastien Bouret ^2^, Rohit Kohli ^1*^

^1^ Gastroenterology, Hepatology and Nutrition, ^2^ Developmental Neuroscience Program & Diabetes and Obesity Program, Center for Endocrinology, Diabetes and Metabolism, ^3^ Pathology and Laboratory Medicine, Children’s Hospital Los Angeles & University of Southern California Keck School of Medicine, Los Angeles, CA 90027

^#^ These authors contribute to the article equally

^*^ Corresponding author: Rohit Kohli. Division of Gastroenterology, Hepatology and Nutrition, Children’s Hospital Los Angeles & University of Southern California Keck School of Medicine. 4650 Sunset Blvd, MS 78, Los Angeles, CA 90027. Phone: (323)361-5924. Email: [rokohli@chla.usc.edu](mailto:rokohli@chla.usc.edu).

Short tile: Rebaudioside ameliorates NASH

**Supplementary Data:**

**
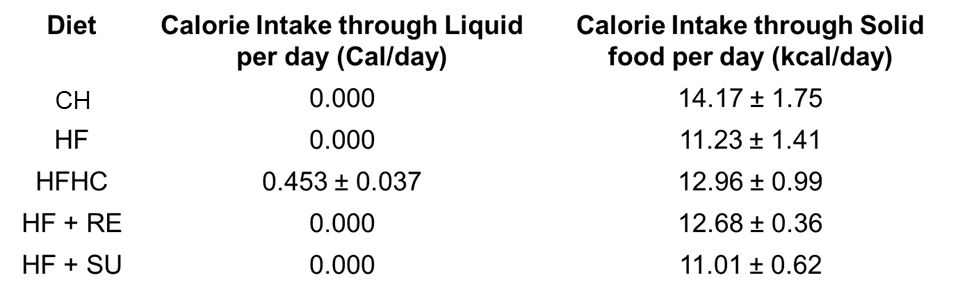
**

**Supplementary Table 1.** Calorie intake through liquid and solid food per day in each group.

**
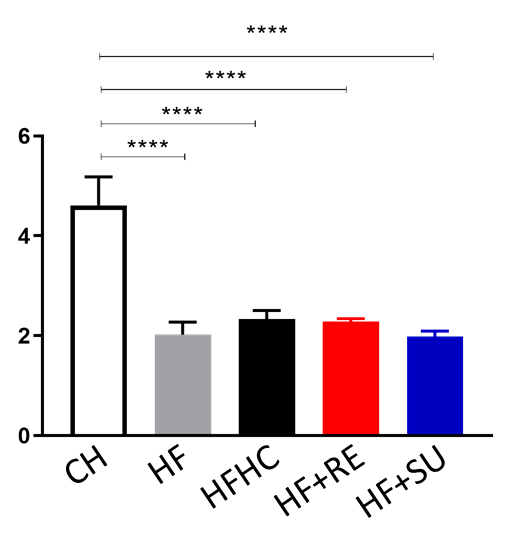
**

Solid Food intake (gram/day)

**Supplementary Figure 1.** Solid Food intake of each experimental group (gram/day).

**
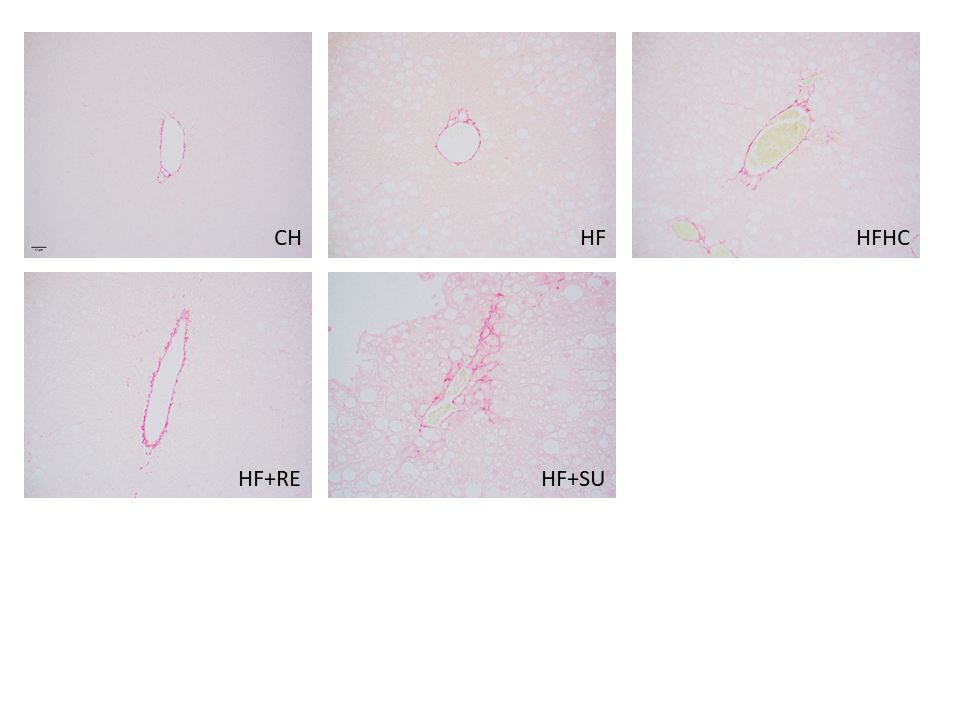
**

**Supplementary Figure 2.** Representative images of Sirius Red stained of mice liver tissue of each experimental group at 400X magnification displaying hepatic fibrosis.

**Immuno blot of CHOP using liver lysate of the animals.**

**Methods and materials:**

Liver protein lysate prepared from ~20mg of mice liver tissue (n=6 for treated groups; n=2 for CH group) using RIPA buffer (Thermo Sceintific, Waltham, MA) containing Protease/Phosphatase inhibitor cocktail (Thermo Sceintific, Waltham, MA). Lysate was centrifuged at 12000g at 4^o^C for 30 minutes and supernatant was used to determine the protein concentration using BCA Assay (Thermo Sceintific, Waltham, MA). 50µg protein lysate mixed with 4X Laemmli Reduced Buffer and heated at 100^o^C for 5 minutes prior to loading in NuPAGE 4-12% Bis-Tris Protein Gels, 1.0 mm, 15-well (Thermo Sceintific, Waltham, MA). iBlot Transfer Stack (Thermo Sceintific, Waltham, MA) was used to transfer protein on nitrocellulose membrane. The membrane was incubated with Odyssey Blocking Buffer TBS (Licor, ‎Lincoln, NE) at room temperature for 1 hour. The membrane was given three times wash, 5 minutes each, using Tris Buffered Saline containing 0.1% Tween-20 (TBST). The membrane was incubated with anti-CHOP (L63F7) Mouse mAb (Cell Signaling Technology, Danvers, MA) (Citation: <https://www.ncbi.nlm.nih.gov/pubmed/31286669>) and β-Actin (13E5) Rabbit mAb (Cell Signaling Technology, Danvers, MA) (Citation: <https://www.ncbi.nlm.nih.gov/pubmed/31432137> ) overnight at 4^o^C. The membrane was given three times wash, 5 minutes each, using TBST. The membrane was incubated with IRDye 680RD Goat anti-Mouse IgG (H + L) (Licor, ‎Lincoln, NE) and IRDye 800CW Donkey anti-Rabbit IgG (H + L) (Licor, ‎Lincoln, NE) for 1 hour in room temperature. The membrane was given three times wash, 5 minutes each, using TBST. The membrane was viewed using LICOR ODYSSEY 9120 - IMAGING SYSTEM (Licor, ‎Lincoln, NE) and the densitometry of CHOP and β-Actin expression was determined using Image Studio Software version 5.2.5 (Licor, ‎Lincoln, NE).

Result:

We observed a trend in increase of CHOP protein in the liver of mice from HFHC group in comparison to that of HF+RE and HF+SU groups. (CHOP: 1.44±0.47 (HFHC) vs 1.09±0.28 (HF), 0.78±0.10 (HF+RE), 0.77±0.06 (HF+SU), 0.62±0.04 (CH). (Supplementary Figure 2-6)

**
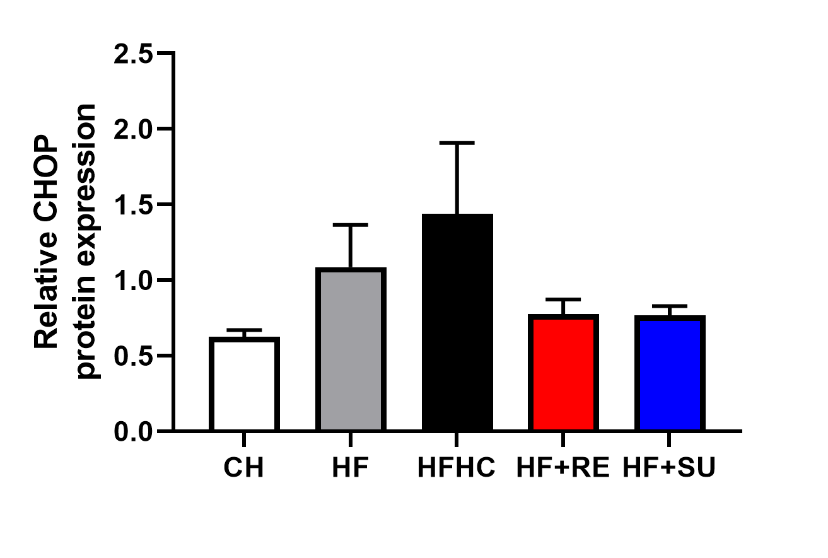
**

**Supplementary Figure 3.** Densitometry plot of CHOP expression in the liver of mice from (i) Chow (CH), (ii) HF (HF+water), (iii) HFHC, (iv) HF+RE (HF+Stevia) and (v) HF+SU (HF+Sucralose).


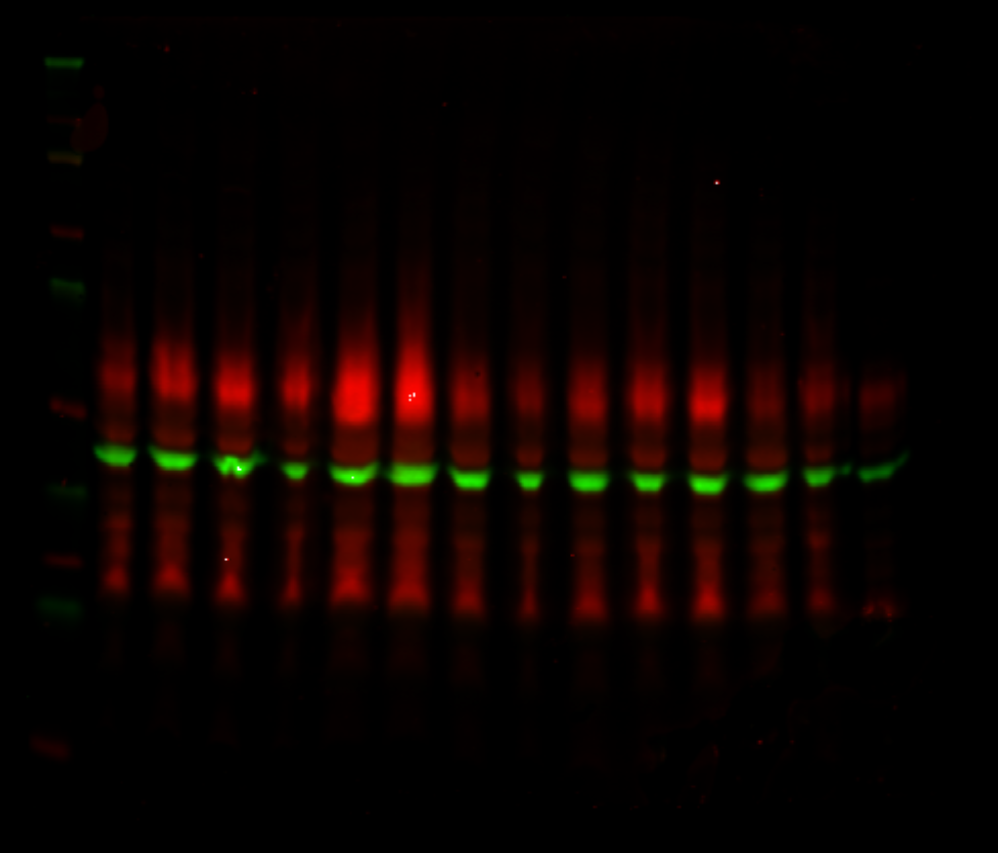


**Supplementary Figure 4.** Image of whole immuno-blot. CHOP was detected using IRDye 680RD Goat anti-Mouse IgG (H + L) (RED) and β-Actin was detected using IRDye 800CW Donkey anti-Rabbit IgG (H + L) (GREEN). Chameleon Duo Pre-stained Protein Ladder for Western Blots was used.


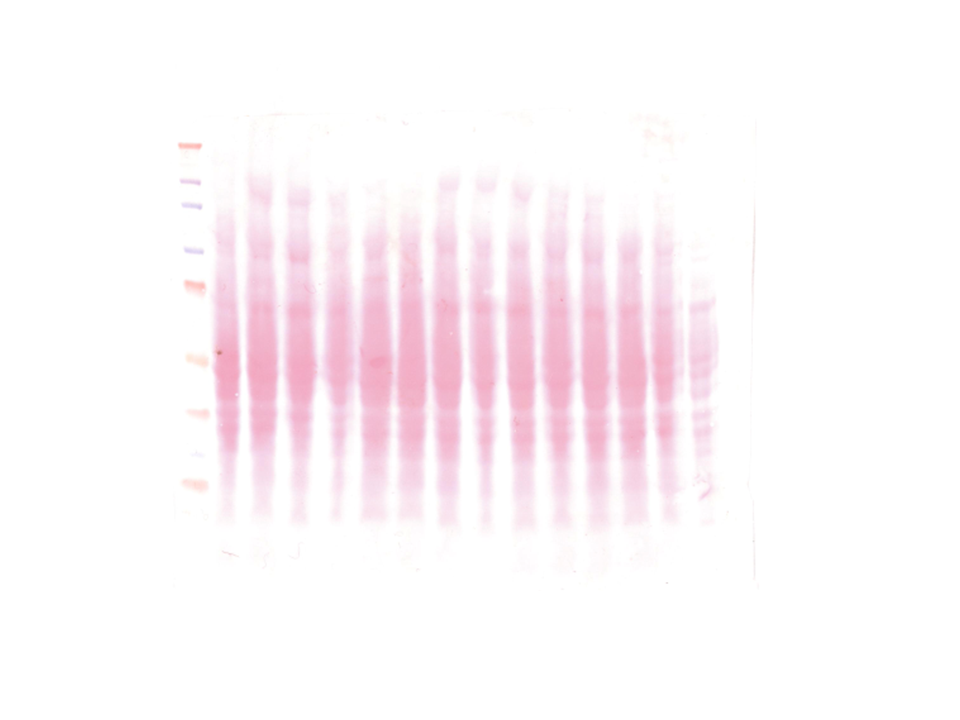


**Supplementary Figure 5.** Image of Ponceau S stain of whole Image Studio Lite ver 5.2.5, Licor, ‎Lincoln, NE). immuno-blot. Chameleon Duo Pre-stained Protein Ladder for Western Blots was used in Lane 1.


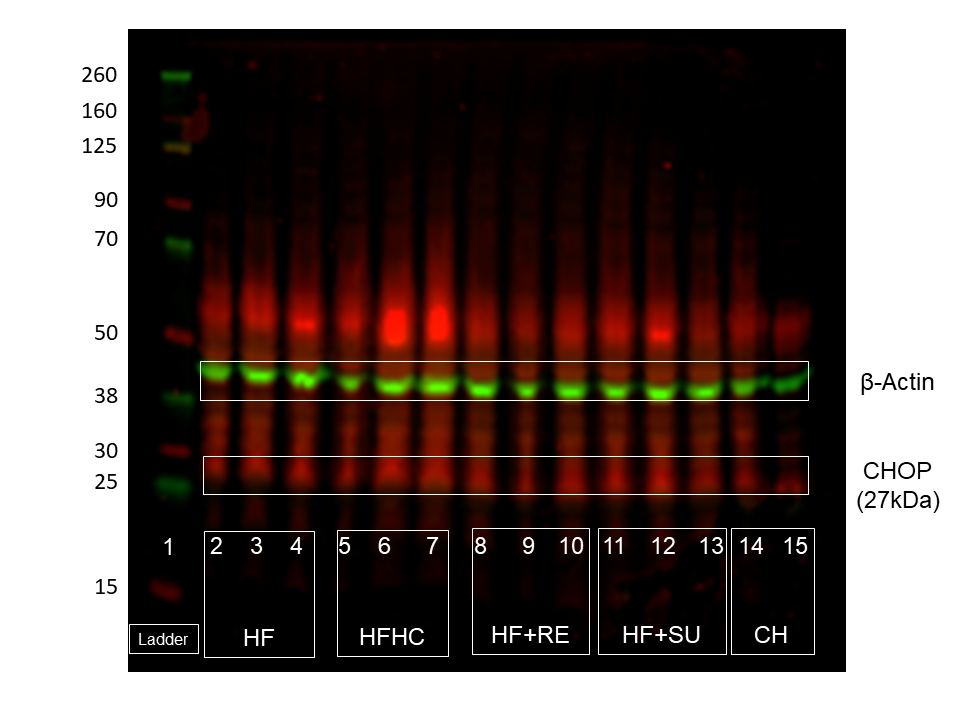


**Supplementary Figure 6.** Image of whole immuno-blot with lane description. CHOP was detected using IRDye 680RD Goat anti-Mouse IgG (H + L) (RED) and β-Actin was detected using IRDye 800CW Donkey anti-Rabbit IgG (H + L) (GREEN). Chameleon Duo Pre-stained Protein Ladder for Western Blots was used in Lane 1.

**Supplementary Figure 7.** Densitometry analysis of immuno-blot of CHOP (27kDa) normalized with β-actin (45kDa) for each sample
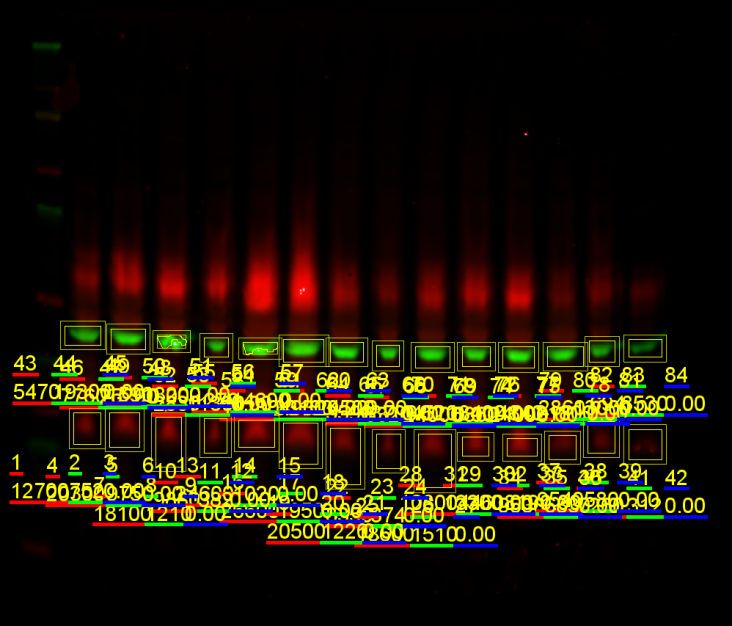
 using Image Studio Lite version 5.2.5 (Licor, ‎Lincoln, NE).
